# Evaluation of Laboratory Findings of Patients with Coronavirus Disease 2019 in Kerman, Iran

**DOI:** 10.30699/IJP.2023.1971332.3031

**Published:** 2023-07-16

**Authors:** Parisa Khorasani Esmaili, Shahriar Dabiri, Sajjadeh Movahedinia, Saeedeh Shojaeepour, Fatemeh Bagheri, Hanieh Ranjbar, Manzumeh Shamsi Meymandi, Elham Mohebbi, Mehrdad Farrokhnia

**Affiliations:** 1 *Department of Pathology, Pathology and Stem Cells Research Center, Afzali Pour Medical Faculty, Kerman University of Medical Sciences, Kerman, Iran*; 2 *Department of Basic Sciences, Faculty of Veterinary Medicine, Shahid Bahonar University of Kerman, Kerman, Iran*; 3 *Legal Medicine Research Center, Legal Medicine Organization, Kerman, Iran*; 4 *Research Center for Modeling in Health, Institute for Futures Studies in Health, Kerman University of Medical Sciences, Kerman, Iran*; 5 *Infectious and Internal Medicine Department, Afzalipour Hospital, Kerman University of Medical Science, Kerman, Iran*

**Keywords:** Coronavirus, COVID-19, Laboratory test

## Abstract

**Background & Objective:**

Since December 2019 in Wuhan, China there is a new form of pneumonia and after expansion in other countries, World Health Organization (WHO) called it Coronavirus Disease 2019 (COVID-19). Since the clinical laboratory findings have played an important role in the progression of the disease, this study aimed to evaluate the laboratory findings in COVID-19 patients (before vaccination).

**Methods:**

In this case-control study that was conducted from February to August 2020; the laboratory test status in 101 positive COVID-19 patients was evaluated and compared with 101 healthy individuals.

**Results:**

The results of our study showed that 21% of patients had low WBC, 24.75% low RBC, 37.62%, low Hb, 18.81% with low HCT, 29.7%, low Plt, 41.58% had High PT, 71.29% high CRP, 17.82% high urea, 11.88% high CR, 15.84% high LDH, 10.89% low sodium, 14.75% low potassium (K). The quantitative examination of blood factors showed that lymph%, mixed%, PLT, HCT, Hb, and RBC were higher in the control group than in the case group. While Neu%, WBC, PTT, CRP, UREA, LDH, K in the patient group were higher than in the control group.

**Conclusion:**

According to the results of the study, it can be concluded that in the clinical treatment of COVID-19 patients, much attention should be paid to the laboratory indicators to identify and intervene early in critically ill patients.

## Introduction

Since December 2019 epidemic of Coronavirus Disease 19 (COVID-19) worldwide, studies are started on this virus. This kind of Coronavirus was isolated from human airway epithelial cells, and it can cause severe acute respiratory syndrome COV2 (1). In the past two decades after severe acute respiratory syndrome coronavirus (SARS-COV) and Middle East respiratory syndrome coronavirus (MERS-COV), SARS-COV2 is the third virus from this group that treatment public health around the world (1-5).

Complete viral genome analysis shows 89.1% nucleotide similarity to a group of SARS-like coronaviruses (genus Betacoronavirus) (6-12).

The mean incubation period was 5.2 days. Studies show human-to-human transmission has occurred among close contacts (13, 14). Common clinical symptoms include fever, cough, tiredness, dyspnea, and loss of taste and smell. Less common symptoms are sore throat, headache, diarrhea, and chest pain (5).

The gold standard for diagnosis of COVID-19 is the identification of viral genome with PCR in respiratory tract secretion during the first week of symptoms (6). Mortality rate increased in older age, with high Sequential Organ Failure Assessment (SOFA) score and when d-dimer greater than 1 μg/mL (on admission) (15-18).

COVID-19 infection leads to many abnormal laboratory parameters. Hematological, biochemical, and coagulation-fibrinolysis tests are adequate indicators. Therefore, the evaluation of clinical laboratory parameters can help to stop disease progression, decrease the side effects of the virus, and can help to diagnose patients in an early stage of COVID-19 infection before showing positive PCR tests. 

This study aimed to determine and compare the significant differences in laboratory tests (CBC and biochemistry) between the COVID-19 patients with healthy control groups (normal populations before the COVID-19 pandemic) in Kerman, Iran.

## Material and Methods

This case-control study’s population consisted of 101 patients with COVID-19 infection, which were confirmed by polymerase chain reaction (PCR) test (according to WHO (World Health Organization) guidelines) and admitted to Afzalipour Hospital, Kerman, south-eastern Iran, between February and November 2020, in addition to 102 healthy controls that had no disease with the same laboratory exam before COVID-19 pandemic. Included criteria in the study were patients between 20-80 years of age, without a history of using anti-coagulant drugs (such as Warfarin) recently, and no liver or kidney disease. Patients diagnosed with DIC (Disseminated Intra-vascular Coagulation) or increased INR (International Normalized Ratio), PTT (Partial Thromboplastin time), and PT (Prothrombin Test) at the same time were excluded.

**Data Collection**

All patients with COVID-19 infection were confirmed by PCR tests which were performed according to WHO guidelines. Laboratory information was collected from admission files. 

Using the records data about demographic, laboratory tests including CBC, PT, PTT, INR, BUN, Cr, Na, K, BUN, ALK-p, LDH, and CRP on the first day of hospitalization and also for demographic data, habits, lifestyle and past medical history of both groups, questioners were filled.

CBC test included White Blood Cells (WBC) with differentiation including Poly morpho-nuclear (Poly or Neu%) and Lymphocytes, Red Blood Cells (RBC), Hemoglobin (Hb), Hematocrit (HCT), Platelet (PLT) were counted by an automatic cell counter (Sysmex model XP300 Japan).

Biochemical tests such as Lactate Dehydrogenase (LDH), C- Reactive Protein (CRP), Blood Urea Nitrogen (BUN) or Urea, Creatinine (Cr) were measured by auto-analyzer DIRUI CS-400. Sodium (Na), and Potassium (K) were measured by Ion Selective Electrode (ISE – EasyLyte REF2120).

Coagulation function tests, which included Partial Thromboplastin Time (P.T.T), Prothrombin Time (P.T), and International Normalized Ratio (I.N.R), were performed using manual tests and duplicate analysis.

All participants signed a written informed consent, which explicitly provided permission for gathering the relevant clinical data. Those who were unable or unwilling to participate were excluded.

**Statistical** **Analysis**

The STATA (version 14) was used for data analysis.

## Results

This study was a case-control study with 101 hospitalized patients (M: 60%, F: 40%) with PCR-confirmed SARS-CoV-2 infection and 102 control group (M:61%, F:39%). In Table 1 we compared demographic data (such as age, gender, marital status, etc.), habits (like cigarette, opium, alcohol, etc.) and, being at high-risk places such as hospitals, care clinics dorms, hotels, and nursing homes in both control and the patient group. No significant difference in sex or age was observed between the patient (49.46±17.75 years) and the control group (51.03±14.69 years) (*P*= 0.955). Most of the patient and the control group population were married (95% vs. 93%).

Of the patients, 51% were smokers vs. control, which showed only 29% (*P*=0.001). Of the patient group, 7% were water-pipe smokers (control 10%), while 54% used other tobacco (control 34%), 24% were opium addicts (control 20%), and 7% drank alcohol (control 9%) (*P*>0.05). There were no significant differences in opium, alcohol drinking, and being in high-risk palaces between the patient and control group.

Table 2 shows the past medical history of the study population; inpatient group asthma was reported more than in the control group. About other medical histories, there was no significant difference (*P*> 0.005).

Different cancer types in study groups were shown in Table 3. Two patients mentioned a history of lung cancer and one mentioned cervical cancer in the patient group. In the control group pancreas cancer (2 persons), bladder carcinoma (2 persons), laryngeal carcinoma (1 person), and 1 person mentioned cervical cancer were reported.

**Table 1:** Demographic data & habits of patient and control groups

Variable	Patient	Control	P-value
Gender	61(60.4% M) 40(39.6% F)	62(60.78% M) 40(39.22% F)	0.955 0.955
Age	53(52.48% are 20-50Y) 48(47.52% are 50-80Y)	51(50% are 20-50Y) 51(50%are 50-80Y)	0.724 0.724
Married	95(94.06%)	93(91.18%)	0.575
Smoking	51(50.5%)	29(28.43%)	0.001
Hookah	7(6.93%)	10(9.8%)	0.460
Tobacco	54(53.47%)	34(33.33%)	0.004
Opium addict	24(23.76%)	20(19.61%)	0.473
Alcohol drink	7(6.93%)	9(8.82%)	0.617
High-risk place	12(11.88%)	8(7.84%)	0.334
Total	101	102	

M means male and F shows female, tobacco means cigarette, hookah, or any type of nicotine.

**Table 2:** Underlying disease in patients and control group**s**

Underlying disease	Patient	Control	P. value
Diabetic type1	5(4.95%)	1(0.98%)	0.5
Diabetic type2(mellitus)	6(5.94%)	5(4.9%)	0.744
Autoimmune disease	0	2(0.96%)	0.157
Cancer/malignancy	3(2.97%)	6(5.88%)	0.314
Hypertension	6(5.94%)	4(3.92%)	0.506
Corticosteroid usage	0	1(0.98%)	0.318
Cardiovascular disease	11(10.89%)	9(8.82%)	0.621
COPD	12(11.88%)	10(9.8%)	0.634
Asthma	17(16.83%)	8(7.84%)	0.051

**Table 3:** Malignancy typing in patient and control groups

Malignancy	Patient	Control
Larynx cancer	0	1
Lung cancer	2	0
Pancreatic cancer	0	2
Bladder cancer	0	2
Cervix cancer	1	1
Total	3	6

Our data about laboratory parameters was arranged and the main laboratory findings were compared in both groups (Tables 4 and 5). Main laboratory tests including WBC, RBC, HB, HCT, PLT, PT, CRP, Urea, Cr, LDH, Na, and K were in the normal range in both patient and control groups, but about 22% of patients show leukopenia (WBC < 4x10^9^ /µL), 25% decrease RBC count and 38% of patients show anemia (Less than 12 gr/dl in Female or less than 14 gr/dL in Male) in Hb concentration.

WBC differentiation also showed a significant difference in the patient and control groups (Neutrophils % 71.34±1.16 and 59.48±0.54), (Lymphocytes % 22.43±0.99 and 29.5±0.47) and (mixed% 5.89±0.41 and 10.54±0.29).

Hematocrit percentage increased only in 5%, thrombocytosis (more than 150-450 x10^9 ^/µL) and thrombocytopenia (less than 150-450 x10^9 ^/µL) were found in 3% and 30% of the patient group, respectively. 

Coagulation tests including PT, PTT, and I.N.R were compared and the analysis shows 52.85% of patients had elevated PT, PTT and it was 41.37 ± 2.34 while in the control group it was 29.29±0.39 (*P*<0.001). INR was 1.26±0.06 in the patient group vs 1.18 ± 0.01 in the control group (*P*<0.001).

Urea in 17.82%, Cr in 11.88% LDH in 15.84% and blood electrolytes Na in 5.94%, and K in 4.95% of the patient group increased significantly (*P*<0.001). C.R.P as an inflammatory factor in blood tests increased in 71% of patients.

**Table 4:** Laboratory parameters in patient and control groups

Laboratory parameter	Patient	Control	P-value
WBC normal count(4-10x10^9^µL )	68(67.33%)	102(100%)	0.001
More than 10x10^9^ µL	11(10.89%)	0	0.001
Less than 4x10^9^ µL	22(21.78%)	0	0.001
RBC normal count(4.2-5.4x10^6^ µL)	74(73.27%)	102(100%)	< 0.001
More than 5.4 x10^6^µL	2(1.98%)	0	< 0.001
Less than4.2 x10^6^ µL	25(24.75%)	0	< 0.001
Hb 12-16 gr/dL in F & 14-18 gr/dL in M	62(61.39%)	102(100%)	< 0.001
More than 16 in F or more than 18 in M	1(0.96%)	0	< 0.001
Less than 12 in F or less than 14 in M	38(37.62%)	0	< 0.001
HCT normal (36-46%)	77(76.24%)	102(100%)	< 0.001
More than 46%	5(4.95%)	0	< 0.001
Less than 36%	19(18.81%)	0	< 0.001
PLT normal (150-450 x10^9 ^/µL)	68(67.33%)	102(100%)	< 0.001
More than 450 x10^9 ^/µL	3(2.97%)	0	< 0.001
Less than 150 x10^9 ^/µl	30(29.7%)	0	< 0.001
PT normal (11-14 seconds )	46(45.54%)	102	< 0.001
More than14s	42(52.58%)	0	< 0.001
Less than 11s	13(12.87%)	0	< 0.001
Positive CRP	72(71%)	0	< 0.001
Urea normal (15-45)	74(73.27%)	102(100%)	< 0.001
More than 45	18(17.82%)	0	< 0.001
Less than 15	9(8.91%)	0	< 0.001
Cr normal	80(79.21%)	102(100%)	< 0.001
More than 1.4 in M or more than1.2 in F	12(11.88%)	0	< 0.001
Less than 0.7	9(8.91%)	0	< 0.001
LDH normal (105-335)	85(84.16%)	102(100%)	< 0.001
More than 335	16(15.84%)	0	< 0.001
Less than 105	0	0	< 0.001
Na normal 135-145	84(83.17%)	102(100%)	< 0.001
More than 146	6(5.94%)	0	< 0.001
Less than 135	11(10.89%)	0	< 0.001
K normal 3.8-5	71(70.3%)	102(100%)	< 0.001
More than 5	5(4.95%)	0	< 0.001
Less than 3.8	25(24.75%)	0	< 0.001

The quantitative examination of blood factors showed that lymph%, PLT, HCT, HB, RBC, and mixed% were significantly higher in the control group than in the patient group. While Neu%, WBC, PTT, CRP, UREA, LDH, and K in the patient group were significantly higher than the control group.

Table 6 shows a significant relationship between smoking, hookah, tobacco usage and COVID-19 infection (*P*<0.005). 

**Table 5:** The laboratory tests range in both patient and control group

Laboratory parameter	Patient range	Control range	P. value	Normal range
WBC	6.6 ± 1.63	5.88 ±2.62	< 0.001	4-10 x10^9^µL
Neutrophil %	71.34±1.16	59.48 ±0.54	< 0.001	40-60%
Lymphocyte %	22.43± 0.99	29.5 ± 0.47	< 0.001	20-40%
Mixed %	5.89 ± 0.41	10.54± 0.29	0.061	5-10%
RBC	4.92 ± 0.07	5.21 ± 0.05	0.001	4.2-5.4 x10^6^ µL
Hb	13.42 ± 0.17	14.72 ± 0.16	< 0.001	12-16 gr/dL in female & 14-18 gr/dL in male
HCT	40.21 ± 0.47	43.71 ± 0.41	< 0.001	36-46%
PLT	195.07 ± 8.92	246.31± 6.97	< 0.001	140-450 x10^9 ^/µL
PT	14.45 ± 0.26	13.97± 0.05	< 0.001	11-14 seconds
PTT	41.37 ± 2.34	29.29 ± 0.39	< 0.001	25-35 seconds
INR	1.26 ± 0.06	1.18 ± 0.01	0.174	Less than 1.5
CRP	40.89 ± 6.10	1.46 ± 0.12	< 0.001	Less than 10mg/L is negative
Urea	33.97 ± 2.66	30.05 ± 0.88	0.157	15-45 mg/dL
Creatinine	1.10 ± 0.07	1.03 ± 0.02	0.368	0.7-1.4mg/L in M and less than 1.2 in F
LDH	415.51 ±23.80	322.16 ± 6.62	< 0.001	105-335 IU/L
Na	139.46 ± 0.46	140.26±0.24	0.123	135-145 mEq/L
K	4.10 ± 0.05	3.88±0.02	< 0.001	3.8-5mEq/L

**Table 6:** Relationship between demographic data, habits, and COVID-19 infection in patient and control group

Parameter	O.R (Odds ratio)	P-value	O.R ( modified)	P-value
Age less than 50y	1		1	
More than 50y	0.9 (0.52-1.57)	0.724	0.94(0.52-1.71)	0.850
Marriage state single	1		1	
Married	1.22(0.36-4.15)	0.744	1.52(0.41-5.60)	0.524
Other	0.40(0.03-5.15)	0.482	0.67(0.04-11.16)	0.786
Smoking No	1		1	
Yes	2.65(1.43-4.57)	**0.001**	6.34(2.51-16.02)	**0.001**
Hookah No	1		1	
Yes	0.68(0.25-1.87)	0.462	0.57(0.18-1.78)	0.339
Tobacco* No	1		1	
Yes	2.29(1.30-4.05)	**0.004**	4.72(1.95-11.38)	**0.001**
Opium No	1		1	
Yes	1.27(0.65-2.49)	0.473	0.53(0.21-1.33)	0.180
Alcohol No	1		1	
Yes	1.27(0.65-2.49)	0.473	0.62(0.19-1.96)	0.643
High risk places No	1		1	
Yes	1.58(0.61-4.05)	0.338	1.27(0.45-3.54)	0.643

*Tobacco include cigarette, hookah or other type of nicotine

In Tables 7 and 8 past medical history and opium intake were evaluated respectively that shows asthma can play a role in predicting underlying disease to COVID-19 infection (*P*=0.036). Also, in COVID-19 patients, last month opium users increased (*P=*0.028).

**Table 7:** The relationship between underlying disease and COVID-19 infection in patient group

Underlying disease	Odd ratio	P-value	Modified Odd ratio	P-value
Diabetic type 1	5.26(0.6-45.84)	0.133	4.46(0.46-43)	0.195
Diabetic type 2	1.22(0.36-4.15)	0.744	2.10(0.54-8.18)	0.282
Malignancy	0.48(0.11-2.01)	0.323	0.41(0.09-1.83)	0.247
Hypertension	1.54(0.42-5.65)	0.509	1.53(0.38-6.10)	0.54
Cardiovascular disease	1.26(0.49-3.19)	0.622	1.24(0.44-3.42)	0.449
COPD	2.41(0.51-3.01)	0.635	1.36(0.52-3.55)	0.528
Asthma	2.37(0.97-5.79)	0.057	2.77(1.07-7.20)	**0.036**

COPD: Chronic obstructive pulmonary disease

**Table 8:** Relationship between times of opium use and infection in the patient group

Opium users in the patient group	Odd ratio	P-value	Modified Odd ratio	P-value
Life type opium user Yes	1.27(0.65-2.49)	0.473	0.59(0.24-1.41)	0.237
Current opium user* Yes	0.31(0.08-1.26)	0.103	0.18(0.04-0.83)	0.028
Regular opium user Yes	0.49(0.15-1.60)	0.240	0.32(0.08-1.18)	0.08

Current opium user*: last month's opium user

## Discussion

Since December 2019, novel coronavirus disease (COVID-19; previously known as 2019-nCoV) has been reported in Wuhan, China, and subsequently affected worldwide. In general, COVID-19 is an acute resolved disease, but it can also be deadly, with a 2% case fatality rate (diffuse alveolar damage) (19). The most common symptoms were fever, shortness of breath, expectoration, fatigue, dry cough, and myalgia (38). Most COVID-19 patients presented with dyspnea with comorbidities such as hypertension and diabetes (39). Our study also showed diabetes, cardiovascular disturbances, respiratory disease including COPD and asthma, and increased blood pressure (hypertension) as comorbidities in COVID-19 patients. Also, asthma increased the hospitalization rate in patients (5).

In a study by Haung *et al.*, most COVID-19 patients were men and underlying diseases include diabetes, hypertension, and cardiovascular disorders. Also, in most of the patients, lymphopenia was detected in the CBC test (20, 21). No significant changes were seen between the patient and the control group (*P*>0.005).

Different studies on laboratory parameters in COVID-19 patients showed significant changes in WBC count; Qin *et al.* (2020), revealed that severe patients of COVID-19 tend to have lower lymphocyte counts, higher leukocyte counts, and neutrophil-lymphocyte ratio (NLR), as well as lower percentages of monocytes, eosinophils, and basophils (mixed). Also, they claimed that the number of T cells significantly decreased. Both helper T (Th) cells and suppressor T cells in patients with COVID-19 were less than normal levels (22). The present study showed that in 21% of patients with low WBC, Neu% in the patient group was significantly higher than in the control group.

In another study, Yuan *et al.* showed lower lymphocyte count, decreased red blood cell and hemoglobin, low levels of immunoglobulin G, and significantly higher D-dimer, fibrinogen, white blood cell count, neutrophil count, interleukin-6, C-reactive protein, procalcitonin, erythrocyte sedimentation rate, ferritin, and lactate dehydrogenase in severe and critically ill patients (23, 24).

Elevated TnT, CRP, and D-dimer, and declined PaO_2_/FiO_2_ suggest that fatality due to COVID-19 was associated with multiple organ dysfunction (23) studies show other coagulation parameters such as PT, PTT, and fibrinogen in non-ICU-patients were less significant in identifying patients needing ICU care (25).

In another study by Badel on 1810 pediatric patient population with positive PCR test for COVID-19 leukopenia, lymphopenia, elevated ferritin, procalcitonin, and CRP was detected in a laboratory study (25-29).

In the transmission of COVID-19, cold and dry weather and low levels of ultraviolet radiation are moderately associated with increased SARS-CoV-2 transmissibility, with humidity playing an important role. Quarantine, physical distancing, and social isolation are effective ways to reduce coronavirus transmission (30-34). There was no significant difference between being in high-risk places such as hotels, care clinics, dorms, and nursing homes between the control and the patient groups.

In multiple studies that analyzed the ACE2 RNA expression in the respiratory tract, ACE2 expression appears in nasal epithelial cells and the size of this population of ACE2-expressing nasal epithelial cells is comparable with the size of the population of ACE2-expression in type II alveolar cells (AT2) (35,36).

In the inflammatory process and cytokines storms IL-19 may play important roles in inflammatory responses because it up-regulates IL-6 and TNF-alpha and induces apoptosis (36).

Aggrawal evaluated the role of TNF-induced apoptosis in T cell deficiency in lymphocytes from aged humans, expression of TNF receptors (TNFRI and TNFRII), and the adapter molecules, including TNFR-associated death domain protein (TRADD), TNFR-associated factor 2 (TRAF-2), and receptor-interacting protein (RIP). An increased constitutive expression of TNFRI and TRADD and decreased expression of TNFRII and TRAF-2 was observed in lymphocytes from aged patients, also increased activation of caspases (caspase-8 and caspase-3) involved in TNFR/TNF signaling pathway (37). Maybe in our study increased CRP levels and lymphopenia can complain with these hypotheses.

Maybe viral infection could be a potential risk factor for idiopathic pulmonary fibrosis because of cytokine storms (38).

Probable reasons for the SARS-associated lymphopenia may be direct infection of lymphocytes by SARS-CoV, lymphocyte sequestration in the lung, or cytokine-mediated lymphocyte trafficking. Also, it could be due to immune-mediated lymphocyte destruction, bone marrow or thymus suppression, or apoptosis (24, 39).

Henry studied 1189 children with COVID-19 infection and showed that monitoring lymphocyte count, CRP, PCT (Procalcitonin), and IL-6 in severe patients would help to manage the treatment of the disease (40). Liu mentioned NLR as an independent risk factor of the in-hospital mortality for COVID-19 patients (more than 6.5) 8% higher risk of in-hospital mortality for each unit increase in NLR and may help identify high-risk individuals with COVID-19 (41).

Hematological factors such as platelet count, prothrombin time, D-dimer, and neutrophil-to-lymphocyte ratio can help clinicians to assess the severity and prognosis of patients with COVID-19. The sepsis-induced coagulopathy scoring system can be used for the early assessment and management of patients with critical disease (42, 43). The result of the present study that used a significant Coagulation test including PT, PTT, and I.N.R showed a significant difference in the patient and the control group (*P* <0.001). It may be because of coagulopathy during sepsis.

## Conclusion

According to the results of the study, it can be concluded that in the clinical treatment of COVID-19 patients, much attention should be paid to laboratory indicators to identify and intervene early in critically ill patients.

## Conflict of Interest

The authors declared no conflict of interest.
